# The Christchurch Earthquake: Crush Injury, Neuropathic Pain, and Posttraumatic Stress Disorder

**DOI:** 10.1155/2013/973234

**Published:** 2013-07-11

**Authors:** Frances Cammack, Edward A. Shipton

**Affiliations:** ^1^Department of Anaesthesia, Christchurch Hospital, Christchurch 8001, New Zealand; ^2^Department of Anaesthesia, University of Otago, P.O. Box 4345, Christchurch 8001, New Zealand

## Abstract

On February 22, 2011, an earthquake of magnitude 6.3 struck Christchurch, New Zealand. The peak ground acceleration, a measure of the shaking or intensity of an earthquake, was one of the highest ever recorded worldwide. One hundred and eighty-five people lost their lives; many others were injured. Two cases both involving young women are presented; they sustained crush injuries to limbs after being trapped by falling debris and went on to develop severe neuropathic pain. This report examines the mechanisms of neuropathic pain in the setting of crush injury, the treatment modalities, and the association between chronic pain and posttraumatic stress disorder. These case reports highlight the fact that crush injury is relatively common during major earthquakes and that neuropathic pain is an important sequel of this. Post-traumatic stress disorder is common in earthquake survivors with a recognised association with chronic pain. Pain-related disability may increase as well. Issues such as chronic pain and physical disability should not be overlooked as attention focuses on disaster management and the treatment of life-threatening injuries.

## 1. Introduction

**Figure 1:**
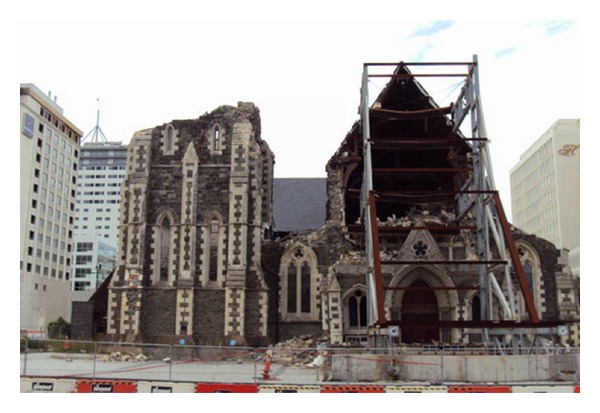
The Christchurch cathedral after the February 22, 2011 magnitude 6.3 earthquake.

On February 22, 2011, an earthquake of magnitude 6.3 struck the city of Christchurch in Canterbury, New Zealand. The peak ground acceleration, a measure of the shaking or intensity of an earthquake, was one of the highest ever recorded worldwide (Figure 1) [1]. 

One hundred and eighty five people lost their lives; many others were injured; the Accident Compensation Corporation (a state insurance company for accidents) received claims from 6659 people [2]. Many injuries were minor, but 142 people required admission to Christchurch Hospital in the first twenty-four hours [2]. 

Two cases both involving young women are presented; they sustained crush injuries to limbs after being trapped by falling debris and went on to develop severe neuropathic pain. This report examines the mechanisms of neuropathic pain in the setting of crush injury, the treatment modalities, and the association between chronic pain and posttraumatic stress disorder (PTSD). Informed consent to submit their case reports for publication was obtained from both patients.

## 2. Case 1

The first patient, a 23-year-old female, was trapped in her workplace for eight hours before being extricated. She sustained severe crush injuries to all four limbs. The left lower leg sustained compound fractures of the tibia and fibula with extensive muscle necrosis and absent distal perfusion. Other injuries received included bilateral fractures of the pubic rami and fractures of the body of S1 and the transverse process of L5. She became critically ill with severe metabolic acidosis (pH 7.05), hyperkalaemia (K^+^ 7.0), and haemodynamic instability that required vasopressor support.

After stabilization, the patient was transferred to the operating room and underwent a left-below-knee amputation and fasciotomies of the right lower leg and both forearms. Bilateral above knee amputations were performed two days later. She subsequently received multiple general anaesthetics for dressing changes, wound closures, and skin grafting.

The patient spent 28 days in intensive care. She initially required ventilator support and dialysis for acute kidney injury secondary to crush injury syndrome. Subsequently, her main problems became pain and stump sepsis. 

Neuropathic pain developed early on in the patient's recovery. She described burning, sharp, and shooting pains in both hands; these pains became progressively worse limiting function. She reported bilateral stump pain with intermittent phantom sensations, as well as phantom pain in her lower legs and feet. 

Pain management was targeted at both nociceptive and neuropathic types of pain. Prescription of analgesia was initially complicated by poor renal function. The analgesic regime consisted of paracetamol, slow release tramadol, gabapentin, venlafaxine, and transdermal clonidine. Parenteral opioids were administered via a fentanyl patient controlled analgesia (PCA) machine; the fentanyl was later converted to oral oxycodone. She was discharged on slow release oxycodone 80 mg bd with immediate release oxycodone for breakthrough pain. A three-day infusion of calcitonin transiently improved the phantom pain. Low-dose ketamine was trialed but abandoned due to hallucinations and flashbacks. 

The patient reported suffering from ongoing severe pain in the left anterolateral thigh above the stump. A left lateral femoral cutaneous nerve block with local anaesthetic gave temporary relief; subsequent neurectomy and burying of this nerve provided more sustained pain relief. 

Signs of posttraumatic stress disorder included anxiety and hypervigilance to loud noises and to sudden movements; ongoing aftershocks were particularly distressing. Her mood fluctuated; her sleep became disturbed. Before the earthquake, she had previously been prescribed citalopram for depression.

Eighteen months after the earthquake, the patient continues to make progress. She reports persistent discomfort in the leg stumps, intermittent phantom pain in her lower legs and feet, and mild neuropathic pain in her hands. Artificial legs were fitted six months after her injury; she mobilises using a self-propelling wheelchair. Her mood has stabilized, and her anxiety has improved. 

## 3. Case 2

The second patient is a 23-year-old female who was on the first floor of a four-storey building when it collapsed. She was trapped for five hours by a large beam; she sustained crush injuries to her pelvis and lower limbs. On arrival at hospital, a crush injury syndrome was diagnosed with resultant severe metabolic acidosis and hyperkalaemia. She was unable to move her lower limbs and reported no sensation below the knees to light touch, pain, or temperature. A CT scan of the pelvis showed bilateral fractures of the superior and inferior pubic rami with distraction of the pubic symphysis. There were no spinal injuries and no lower limb fractures. 

Due to the high demand on the local intensive care service, the patient was transferred to another hospital. She remained in intensive care for two weeks and required dialysis for her crush injury syndrome. 

There was profound weakness of  both legs for a week after the injury; this then slowly improved, although persistent patchy numbness and a right foot drop remained. The patient developed neuropathic pain in the right foot soon after the injury that was described as burning and stinging in the right sole. The pain was constant and severe (reaching 9/10 to 10/10 on a verbal rating scale). It was accompanied by allodynia; even light touch felt exquisitely painful. She could not tolerate physiotherapy; she required a 50% nitrous oxide/50% oxygen combination inhalation for splint application. The pain spread to involve the left foot as well. 

The neuropathic pain and allodynia slowly reduced in intensity; the severe flareups, however, persisted. Autonomic features developed; these included a dusky appearance of the right foot accompanied by mild swelling, decreased sweating, and abnormal skin texture. Electromyography and nerve conduction studies demonstrated a severe lesion of the right sciatic nerve.

The patient's analgesic regime consisted of paracetamol, gabapentin, amitriptyline, tramadol, transdermal clonidine, and a fentanyl PCA. The patient was later converted to a fentanyl patch with oral immediate release oxycodone as needed. Ketamine was trialed early but caused unpleasant hallucinations and was stopped. An intravenous low-dose lignocaine infusion proved ineffective. 

In hospital, the patient experienced low mood as well as some features of PTSD. On discharge, symptoms such as flashbacks and distressing dreams occurred when exposed to cues that triggered recall of events. She did not report significant anxiety and continued to adjust well. She resumed part-time work eight months after the injury. The neuropathic pain has persisted, particularly in the right foot, but flareups of pain have reduced in frequency and intensity.

## 4. Discussion

Major earthquakes are some of the most devastating natural disasters [3]. They frequently cause numerous victims with a wide variety of injuries [4]. Crush injury is common in this setting; it is estimated to occur in 3–20% of those injured [5]. After a major earthquake, entrapment under collapsed structures and debris accounts for most crush injuries [3]. 

It is known that the shortage of pain-relieving medications (opioid or nonopioid) in the first hours after the earthquake is related to availability and transport [6, 7]. Different forms of pain are under treated. An aggressive attitude in the administration of strong opioids and ketamine should be recommended, so that the eventual onset of chronic pain can be reduced [7]. This should occur alongside aggressive resuscitation measures to provide systemic stabilisation and organ perfusion.

### 4.1. Crush Injury

Crush injury is a form of acute rhabdomyolysis; muscle necrosis, with or without neurological disturbance, occurs after a long period of continuous pressure. The lower limbs are most commonly affected (74%) partly due to the fact that patients suffering prolonged crushing of the torso do not usually survive to reach hospital [8]. 

Crush injury damages tissue by a combination of direct mechanical force and circulatory ischaemia. Muscles can tolerate ischaemia for up to four hours. However, when combined with mechanical force, tissue damage is accelerated, and muscle death can occur within one hour [9]. 

During the revascularisation that occurs once pressure is released from the crushed body part, calcium, sodium, and water diffuse into the necrotic muscle; potassium, lactic acid, myoglobin, and creatine kinase are lost [3, 10]. Acute kidney injury results from impaired perfusion and intratubular obstruction by myoglobin and uric acid [11]. This results in acute renal failure, hyperkalaemia, acidosis, and hypovolaemic shock that can be lethal [3]. 

### 4.2. Neuropathic Pain

Nerve damage after crush injury is characterised by axonal injury followed by Wallerian degeneration. In this process, haematogenously derived macrophages and activated Schwann cells surround the injured axon degrading the myelin sheath. These cells produce growth factors and cytokines creating a favourable environment for regrowth of damaged axons [12]. Wallerian degeneration is likely to be important in the development of neuropathic pain; in the rat model, the timing of this process correlates with the development of hyperalgesia [13]. 

Neuropathic pain arises from damage or pathological change in the peripheral or central nervous system. The mechanism by which crush injury to nerves results in neuropathic pain is complex. It likely involves changes in the peripheral nerve, in the dorsal root ganglion, and in the spinal cord [14]. Immune cells and proinflammatory mediators play an important role. In the rat model, cytokines such as interleukin-1*β* (IL-1*β*) and tumour necrosis factor-*α* (TNF-*α*) are transiently increased around crushed sciatic nerve [15]. Furthermore, intraneural injection of TNF-*α* induces neuropathic pain [16]. Neutrophils, almost absent in uninjured nerves, show significant infiltration around crushed sciatic nerves due to the neural inflammation [17]. In summary, pain may now be considered a neuroimmune disorder; the activation of immune and immune-like glial cells in the dorsal root ganglia and spinal cord gives rise to the release of both pro- and anti-inflammatory cytokines, as well as to algesic and analgesic mediators [18]. 

With neuropathic pain, cytokine interleukins (IL-1alpha, IL-1beta, IL-2, IL-4, IL-6, IL-10, IL-15, and IL-18), tumour necrosis factor-alpha (TNF-*α*), interferon gamma (IFN-gamma), transforming growth factor beta 1 (TGF-beta 1), fractalkine and chemokine C-C motif ligand 2 (CCL2), complement components (C1q, C3, and C5), and metalloproteinases (MMP-2,-9) become activated on spinal cord and dorsal root ganglion levels [18]. TNF-*α* is released by most immune cells, including glial cells located in close proximity of neurones [18]. It can promote neuronal hypersensitivity, increase transmission of excitation, and propagate the ongoing inflammation at multiple levels of the nervous system [19, 20]. TNF-*α* is considered to be the central chronic pain mediator; it may form a promising target for pain treatment [18]. The TNF-*α* monoclonal antibodies (infliximab, etanercept) used in the treatment of human autoimmune diseases (rheumatoid arthritis) have not been studied yet in neuropathic pain [18, 21]. 

### 4.3. Medications

Acute nociceptive pain and neuropathic pain in the crushed limbs were experienced in both case studies. Pain management included primary analgesics to target nociceptive pain such as paracetamol and opioids. Non steroidal anti-inflammatory drugs were contraindicated owing to the acute kidney injury. 

In the absence of evidence for the specific treatment of crush injury-induced neuropathic pain, the patients received the standard antineuropathic pain therapy. Most large randomised controlled trials have focused on patients with painful diabetic neuropathy or postherpetic neuralgia. Recent reviews have gabapentin, pregabalin, tricyclic antidepressants, selective serotonin and noradrenaline reuptake inhibitors (e.g., venlafaxine), and topical lignocaine as first-line agents for the treatment of neuropathic pain [22, 23]. 

Opioid use in neuropathic pain remains controversial. Opioids and tramadol are generally reserved as second-line treatments but become appropriate first-line agents in certain clinical situations such as acute neuropathic pain [22, 23]. Oxycodone was selected as the oral opioid of choice for both patients. Randomised control trials have reported significant benefit of oxycodone over placebo in the treatment of neuropathic pain [24, 25]. The antinociceptive effect of oxycodone is partially mediated by kappa opioid receptors that might contribute to its efficacy in neuropathic pain [26]. 

There is limited evidence that transdermal clonidine is effective in the treatment of neuropathic pain [23]. Intravenous lignocaine has shown efficacy in the treatment of chronic painful diabetic neuropathy [27, 28]. Calcitonin is specific to the treatment of acute phantom limb pain [29] but seems to be ineffective for chronic phantom limb pain [30]. 

The literature examining the association between crush injury and chronic pain is limited. A recent review of medical complications associated with earthquakes omitted to discuss chronic pain [3]. A case series from the Kobe earthquake [31] in Japan discussed two patients with crush injuries who subsequently developed intractable pain. Both described burning pain and allodynia that started one and three weeks after injury, respectively. Epidural analgesia was effective for a short time in one patient; the other responded to a combination of analgesics, transcutaneous nerve stimulation and near infrared radiation. Similar to Case 2, both the Kobe patients transiently lost power and sensation in their lower limbs [31]. 

### 4.4. Recommended Treatment of Neuropathic Pain

But what is the recommended treatment of neuropathic pain? There is a good agreement between guidelines (such as those of the International Association for the Study of Pain Neuropathic Pain Special Interest Group) that tricyclic antidepressants, alpha-2-delta ligands (*α*2-*δ* ligands) (gabapentin and pregabalin), serotonin-norepinephrine reuptake inhibitors (venlafaxine and duloxetine), carbamazepine (for trigeminal neuralgia), and topical lignocaine (for localised peripheral neuropathic pain) are the first-line drugs; tramadol and opioids are second-line drugs [32]. There have only been a few randomised controlled trials that have examined combination therapy; there is a greater pain reduction and fewer side effects when using two drugs together (*α*2-*δ* ligands and opioids, *α*2-*δ* ligands and tricyclic antidepressants) than each drug alone [32, 33].

Other medications can be tried when first- and second-line treatments are ineffective or not tolerated. They include other antidepressants (bupropion, citalopram, and paroxetine), antiepileptic medications (carbamazepine, oxcarbazepine, lamotrigine, phenytoin, topiramate, and valproate), high-concentration capsaicin patches, cannabinoids (for multiple sclerosis), mexiletine, memantine, dextromethorphan, clonazepam, botulinum toxin type A, and intravenous immunoglobulin [32, 34]. With refractory neuropathic pain, invasive treatments (neurostimulation, intrathecal infusion of opioids, local anaesthetics, baclofen, and ziconotide) can be used for patients; the evidence base for these remains weak [32, 35].

There is only limited evidence supporting nonpharmacological treatments in neuropathic pain; these include physical exercise, physical therapies (transcutaneous electrical nerve stimulation and graded motor imagery), cognitive behavioural therapy, or supportive psychotherapy [32]. 

### 4.5. Future Treatment of Neuropathic Pain

Anti-inflammatory interleukins like IL-10, IL-4, IL-1alpha, and transforming growth factor beta 1 (TGF-beta 1) will in future be studied for their effects on attenuating neuropathic pain [18]. Microglia inhibitors will be used for the pharmacological attenuation of glial and immune cell activation. For example, propentofylline, pentoxifylline, minocycline, and fluorocitrate suppress the development of neuropathic pain [18]. Another way of pain control would be to decrease the pronociceptive agents like transcription factor synthesis (nuclear factor kappa-light-chain-enhancer of activated B cells, activator protein 1), kinase synthesis (p38 Mitogen-activated protein kinases, c-Jun N-terminal kinase), and protease activation (cathepsin S, matrix metallopeptidase 9, matrix metallopeptidase 2) [18]. Research is being carried out in order to replace current pharmacological treatments for neuropathic pain with cellular therapies (such as the use of stem cells) with more lasting effects [36]. 

### 4.6. Posttraumatic Stress Disorder

Posttraumatic stress disorder (PTSD) is a condition where symptoms evolve in the aftermath of an extreme traumatic stressor that overwhelms the individual's coping capacities [37]. The psychological impact of an earthquake is significant; survivors of severe earthquake trauma have an incidence of posttraumatic stress disorder (PTSD) as high as 87% [38, 39]. Women are more likely than men to experience PTSD [40] as are those with physical injuries [39]. Specific to earthquakes, ongoing aftershocks serve as a frequent reminder of the initial trauma; this leads to a reexperiencing of the event, to more hypervigilance, and to more anxiety. To date, in Christchurch, there have been more than four thousand aftershocks exceeding magnitude three on the Richter scale [41]. 

An association exists between posttraumatic stress disorder and chronic pain [42, 43]. The most common types of chronic pain include pelvic pain, lower-back pain, facial pain, and bladder pain, along with a high prevalence of fibromyalgia [42, 43]. Another example is that 75% of patients with chronic headache after a motor vehicle accident experience symptoms consistent with PTSD as well [44]. 

It has been proposed that chronic pain and PTSD share a number of common factors that result in their coexistence [45]. For example, anxiety, which in itself is a key feature of PTSD, increases pain perception. Pain may serve as a persistent reminder of the traumatic event; it may trigger an arousal response leading to avoidant behaviour. Avoidance of painful activities results in physical deconditioning and increased disability.

PTSD and chronic pain form a high comorbidity with depression [46]. Patients with depression experience increased pain morbidity; they report more severe pain intensity and greater pain-related disability and are more likely to suffer from poor treatment outcomes compared to those who are not depressed [47]. 

## 5. Conclusion

These case reports highlight the fact that crush injury is relatively common during major earthquakes; neuropathic pain is an important sequel of this. Alongside aggressive resuscitation measures, an aggressive attitude in the administration of strong opioids and ketamine is recommended, so that the eventual onset of chronic pain can be reduced [7]. 

PTSD is also common in earthquake survivors. It has a recognised association with chronic pain. Pain-related disability may increase with time. Issues such as chronic pain and physical disability may be overlooked as attention focuses on disaster management and the treatment of life-threatening injuries.
